# 3-Deoxysappanchalcone attenuates LPS-induced neuroinflammation in microglia cell culture and ameliorates cognitive impairment in traumatic brain injury

**DOI:** 10.1371/journal.pone.0323259

**Published:** 2025-05-30

**Authors:** Zemeng Li, Dangli Ren, Jingjing Wang, Yatao Wang, Yueyang Chen, Yunfeng Diao, Jianwei Li, Yang Qu, Maohua Zheng, Hongtao Sun

**Affiliations:** 1 Tianjin Key Laboratory of Neurotrauma Repair, Institute of Traumatic Brain Injury and Neuroscience, Characteristic Medical Center of Chinese People’s Armed Police Force, Tianjin, China; 2 The First Clinical Medical College of Lanzhou University, Lanzhou, China; Nationwide Children's Hospital, UNITED STATES OF AMERICA

## Abstract

**Background:**

As one of the major public health security problems, traumatic brain injury (TBI) is characterized by cerebral dysfunction. The following neuroinflammation is considered as the main secondary injury factor. Targeting the expression of inflammatory cytokines could be effective in alleviating TBI-induced neuroinflammation. The anti-inflammatory role of natural products is increasingly receiving attention. 3-Deoxysappanchalcone (3-DSC) is a bioactive compound from Caesalpinia sappan L.

**Methods:**

The present study was designed to investigate the impact of 3-DSC on neuroinflammation in primary microglia and TBI models. To assess cytotoxicity, cell viability tests were conducted with varying concentrations of 3-DSC ranging from 5 to 20 μM. Quantitative PCR (qPCR) and Enzyme-Linked Immunosorbent Assay (ELISA) were utilized to measure the production of inflammatory cytokines in LPS-activated primary microglia treated with or without 3-DSC (at 10 μM). Immune blotting arrays were used to examine the activation of canonical inflammation signaling pathways. To further elucidate the anti-inflammation effect of 3-DSC, RNA-seq was carried out between LPS and LPS + 3-DSC group. In vitro co-culture experiments were carried out to evaluate the protective effect of 3-DSC on neurons against inflammation-mediated apoptosis. Additionally, in vivo experiments were performed to observe the impact of 3-DSC on TBI-induced microglia activation and spatial memory impairment. 3-DSC (160 μg/kg, 320 μg/kg) were administered via the tail vein at day 1 after TBI (n = 6). Behavioral tests were conducted 7 days after traumatic brain injury (TBI) to detect the spatial memory ability of rats.

**Results:**

The cell viability results revealed that within the concentration range of 5–20 μM, 3-DSC did not cause significant cytotoxicity. In the qPCR and ELISA assays, it was found that 3-DSC at 10 μM led to a reduction in the production of inflammatory cytokines. The immune blotting arrays demonstrated that 3-DSC inhibited the activation of NF-kB and MAPK signaling pathways. The results of RNA sequencing revealed the altered signaling pathways and key hub genes. The in vitro co-culture outcomes indicated that 3-DSC could safeguard neurons from apoptosis caused by neuroinflammation. Finally, the in vivo experiments showed that 3-DSC was effective in alleviating TBI-induced microglia activation and spatial memory impairment.

**Discussion:**

Collectively, these findings suggest that 3-DSC holds promise as a potential compound for the development of therapeutic and preventive agents aimed at treating neuroinflammation-related disorders. It offers a new avenue for further research and potential clinical applications in the context of TBI and neuroinflammation related disorders.

## Introduction

Traumatic brain injury (TBI) is a significant global public health concern, affecting over 50 million individuals annually, thus imposing substantial burdens on both families and society [1]. Resulting from external force impact, TBI manifests as cerebral dysfunction. Subsequent microglia-mediated neuroinflammation is identified as the primary secondary injury factor, potentially culminating in neurodegeneration and other brain-related disorders. [2]. Numerous studies underscore the pivotal role of inflammatory mediators, including tumor necrosis factor (TNF), interleukin (IL)-1β, and interleukin (IL)-6, in orchestrating the progression of TBI [3–5]. Targeting microglia and attenuating the expression of inflammatory cytokines emerge as promising strategies to mitigate TBI-induced neuroinflammation.

3-Deoxysappanchalcone (3-DSC) is indeed a chalcone-based chemical compound derived from Caesalpinia sappan L. (C. sappan), a plant belonging to the Leguminosae family [6]. Caesalpinia sappan L. has garnered interest due to its potential medicinal properties in improving blood circulation [7]. Compounds extracted from C. sappan have been shown various biological activities, including anti-influenza, anti-inflammatory, anti-oxidation, anti-allergy, immunomodulation effect [8–10]. According to previous reports, the anti-cancer activity of 3-DSC was confirmed in multiple cancers including esophageal cancer and colon cancer [11,12]. However, there is not yet enough evidence to prove the anti-inflammation activity of 3-DSC in microglia.

Based on the current knowledge, we aimed to investigate the possible roles of 3-DSC in neuroinflammation. To explore the role of 3-DSC in neuroinflammation, we established in-vitro neuroinflammation models, coculture models and in-vivo TBI rat models. In our study, we found that 3-DSC had the ability of inhibiting the production of inflammatory cytokines in in-vitro neuroinflammation models. The classic inflammation signaling pathways, including NF-κB and MAPK signaling pathways, were both inhibited by 3-DSC in the in-vitro models. Excessive neuroinflammation can lead to neuronal apoptosis. Protecting neurons from apoptosis also plays an important role in the treatment of neuroinflammation. The in-vitro coculture assay was carried to mimic the neuron status during neuroinflammation. The results showed that 3-DSC could protect neurons from neuroinflammation-mediated apoptosis. In TBI rat models, injection of 3-DSC via tail vein can alleviate TBI mediated microglia activation and cognitive decline, moreover, the levels of inflammatory factors in brain tissues decreased. Our study will provide a better insight into the function of 3-DSC and may lead to a promising new treatment for neuroinflammation.

## Materials and methods

### Antibodies and regents

Antibodies against NF-κB p65 (8242), Phospho-NF-κB p65 (3033), p38 MAPK (8690), Phospho-p38 MAPK (4511), PARP (9532), Cleaved Caspase-3 (9664), TNF-α (11948), IL-6 (12912) were from Cell Signaling Technology. Antibodies against IL-1 beta (AF-401-NA) were from R&D. Antibodies against GAPDH (60004–1-Ig), IBA1 (26177–1-AP) were from Proteintech. INTERFERin (409−10) reagent were from Polyplus Transfection. PrimeScript™ RT Master Mix (RR036A), TB Green® Premix Ex Taq™ II (RR820A) was from Takara. Mouse TNF Alpha ELISA Kit (abs520010), Mouse IL-6 ELISA Kit (abs520004) and Mouse IL-1 Beta ELISA Kit (abs520001) were from Absin Bioscience. Cell Counting Kit-8(CK04) were from Dojindo. LPS (L2630) were from Sigma. Nuclear and Cytoplasmic Protein Extraction kit was purchased from Beyotime (P0028-1). Dead Cell Apoptosis Kit with Annexin V FITC & Propidium Iodide(V13241) were from Thermofisher. 3-Deoxysappanchalcone (HY-N1745A) were from MCE.

### Extraction of primary microglia and neuron and cell culture

Primary cortical neurons were obtained from 1-day-old C57BL/6J mice [13]. The mice were sacrificed, and then the brain was placed in pre-cooled Dulbecco’s modified Eagle’s medium/nutrient mixture F-12 (DMEM/F12) medium. Then the dissected cerebral cortex was digested with 0.25% trypsin and DNase at 37 °C for 20 min. The digestion was filtered through the cell strainer. Then, the filtrate was centrifuged at 1000 rpm for 5 min, and the supernatant was discarded. The cell pellet was resuspended in DMEM/F12 medium containing 10% horse serum and 2% penicillin and streptomycin (Gibco). After about 4 h, the medium was replaced with neurobasal medium containing 2% B27 and 0.5 mM glutamate, and the dishes were placed in a cell incubator at 37 °C, with 5% CO2.

The preparation of primary microglia was similar to that of neurons [14]. The difference was the medium. DMEM/F12 for microglia contained 10% fetal bovine serum, 1 mM sodium pyruvate, 2 mM L-glutamine, 100 mM nonessential amino acids, 50 U/mL penicillin, and 50 mg/mL streptomycin (all from Gibco). After plating, cells were cultured for 2 days in a poly-D-lysine pretreated 150-cm2 culture flask with. Within 2 weeks of seeding, glial cells formed a confluent monolayer. Microglia were separated by shaking and were enriched by centrifuging.

### RNA isolation and quantitative real-time RT-PCR

Total RNAs were extracted using Cell Total RNA Isolation Kit (Foregene). The complementary DNA was performed using the PrimeScript™ RT Master Mix (Takara) according to the manufacturer’s instructions. The expression of genes was measured by using ABI Life QuantStudio 6 with TB Green Real time PCR Master Mix (Takara) according to the manufacturer’s direction. All the samples were normalized to β-actin according to the 2^-ΔΔCT^ method. All primers used in this study were synthesized by Sangon Biotech. The following primers were used:

**Table:** 

Gene	Forward primer sequence (5′–3′)	Reverse primer sequence (5′–3′)
β-actin	GGCTGTATTCCCCTCCATCG	CCAGTTGGTAACAATGCCATGT
IL-6	CTGCAAGAGACTTCCATCCAG	AGTGGTATAGACAGGTCTGTTGG
IL-1b	CAGGCTCCGAGATGAACAAC	GGTGGAGAGCTTTCAGCTCATA
TNFA	CAGGCGGTGCCTATGTCTC	CGATCACCCCGAAGTTCAGTAG
Ifit1	GCCTATCGCCAAGATTTAGATGA	TTCTGGATTTAACCGGACAGC
Ifit2	GGAGAGCAATCTGCGACAG	GCTGCCTCATTTAGACCTCTG
Ifit3	GAAGGAAGTATGTCC	GGTAGATAGACCACGAAAT
Cd40	TGTCATCTGTGAAAAGGTGGTC	ACTGGAGCAGCGGTGTTATG
Ccl2	TTCTTCGATTTGGGTCTCCTTG	GTGCAGCTCTTGTCGGTGAA
Irf1	ATGCCAATCACTCGAATGCG	CCTGCTTTGTATCGGCCTGT
Cd86	TCAATGGGACTGCATATCTGCC	GCCAAAATACTACCAGCTCACT
Dhx58	GGAAGTGATCTTACCTGCTCTGG	TTGCCTCTGTCTACCGTCTCT

### Conditioned media (CM) assays

For CM collection, primary microglia cells were pretreated with 3-Deoxysappanchalcone (10 μM) for 1h and then were treated with LPS (1 μg/ml) for 8 h. The CM from different treatment groups was collected and centrifuged at 12,000 g for 10 min at 4°C. Primary neurons were cultured with CMs for 24 h, and cell viability was then analyzed.

### Flow cytometer assay

Primary neurons were seeded in a 12-well plate and were stimulated with CM for 24h, then subjected to Annexin V and PI (ThermoFisher, V13241) staining. After incubation, cells were harvested and washed with PBS and then resuspended in 500 μL PBS. Cell fluorescence was acquired on a Beckman Coulter DxFLEX flow cytometer and analyzed with FlowJo software. The Annexin V positive cells were regarded as apoptotic cells.

### Nuclear and cytoplasmic protein extraction

Nuclear and cytoplasmic Protein were extracted according to the manufacturer’s instructions (Beyotime. P0028-1). Microglia were washed with ice cold PBS and resuspended in 200 μL ice-cold cell lysis buffer. After sitting on ice for 15 min, cell lysates were spun in a microcentrifuge at 12000 g for 5 min at 4 ◦C and supernatants were aliquoted and stored at − 80 ◦C for Western blot analysis. Nuclear pellets were then washed in 500 μL of cell lysis buffer and resuspended in 150 μL of nuclear extraction buffer. After vigorously shaking at 4 ◦C for 30 min, nuclear extracts were aliquoted and stored at − 80 ◦C until use.

### Western blot analysis

The total proteins were extracted with Cell lysis buffer (Cell Signaling Technology) containing PMSF (Cell Signaling Technology) and quantified with BCA protein assay kit (Thermo Fisher Scientific). The proteins were separated by SDS-PAGE (LEAGENE) and transferred to the NC membranes. After washing with TBST (Solarbio) and blocking with 5% BSA, the membranes were subjected to the specific primary and secondary antibodies incubation. Detection of proteins was imaged using ImageQuant LAS 4000 mini (GE Healthcare Life Sciences).

### Animals

Male Sprague Dawley rats (SD rats), weighing between 230–250 grams, were sourced from spfbiotech (Beijing). These rats were individually housed in cages placed in a room where the temperature was maintained at 22 ± 1˚C. The animals were kept under a standard 12 – hour light and 12 – hour dark cycle and had unrestricted access to food and water.

After TBI modeling and behavioral experiments (as below), rats underwent anesthesia through an intraperitoneal injection of pentobarbital sodium at a dosage of 50 mg/kg. Once the anesthesia took full effect and the rats showed no signs of responsiveness, an additional lethal dose of pentobarbital sodium was administered intraperitoneally to ensure euthanasia. The lethal dose was calculated based on the rats’ body weight, typically around 100–150 mg/kg for a complete cessation of physiological functions. Subsequently, 0.01 M phosphate - buffered saline (PBS) was used for intracardiac perfusion. This was then succeeded by intracardiac perfusion with 4% paraformaldehyde (PFA) dissolved in PBS. The brain was removed and the brain tissues around the damaged region were collected for further immunofluorescence and western blot analysis.

### TBI model construction and 3-Deoxysappanchalcone injection

TBI was induced in SD rats using a cortical contusion injury (CCI) model [15,16]. Rats were anesthetized by an intraperitoneal injection of pentobarbital sodium (50 mg/kg) and placed in the stereotaxic frame. A 4 mm-diameter craniotomy was performed using a portable drill over the right parietal cortex between bregma and lambda, 2 mm lateral to the midline. The dura mater was kept intact over the cortex.

The CCI was performed perpendicular to the brain surface using a cortical Impactor (VCU-CDF, USA) with a 3-mm-diameter impact tip. The impact velocity is 3 m/s, the impact duration is 150 ms, and the impact depth is 2 mm. Aft er TBI, the bone flap was immediately replaced, and the scalp was sutured closed. Sham animals were subjected to all aspects of the protocol (surgery, anesthesia, craniotomy, injection, and recovery) except for CCI. 3-Deoxysappanchalcone (160 μg/kg, 320 μg/kg) were administered via the tail vein at day 1 after the CCI operation.

### Immunofluorescence and image analysis

Rats were anesthetized and transcranial perfused with 0.1 mmol PBS and 4% paraformaldehyde (PFA) at 7 days post-TBI. Twenty-micrometer coronal cryosections were permeabilized and incubated in 5% Donkey Serum for 1 h for blocking. Then the brain tissues were incubated in Iba1(26177–1-AP, Proteintech) primary antibody overnight at 4 °C. After the incubation overnight, the cryosections were incubated with the secondary antibodies for 1 h at room temperature. After that, the sections were rinsed with PBS and covered with fluorescence mounting medium with 4’,6-diamidino-2-phenylindole (DAPI) (Vector Laboratories, H-1200).

For image analysis and quantification of immunofluorescent. In each animal, 3 randomly selected fields from 3 nonadjacent sections were analyzed. For immunofluorescence analysis, the fluorescence intensity of Iba-1 was calculated by using ImageJ software.

### Behavior test

Morris water maze experiment is a commonly used method to assess spatial learning and memory capabilities. The experimental procedures are consistent with those reported in previous literature [17]. Briefly, the experiment was carried in a circular water tank with a diameter of 1.6 meters. The tank was filled with water at approximately 22°C and rendered opaque with water-soluble, non-toxic white paint. A small platform with a diameter of 18 cm was placed in the water for the rats to find. The learning phase lasted 5 days, with training occurring 4 times each day. The rats were trained to locate the hidden platform. In the memory phase, the platform was removed. Memory retention of rats was assessed based on the number of times they passed through the area where the platform had been removed.

The mNSS (modified Neurological Severity Score) is a scale used to assess neurological deficits. It is commonly used to evaluate neurological impairments in brain injury or neurological disease models. The mNSS typically includes the following aspects to comprehensively assess the degree of neurological impairment: motor function, sensory function, reflexes, and behavioral observations. The score ranges from 0 to the maximum value, with higher scores indicating more severe neurological deficits [18].

### Ethics approval and consent to participate

The animal study was reviewed and approved by the Animals Care and Use Committees of the Institute of Laboratory Animal Sciences of Chinese Academy of Medical Sciences (ACUC-A01-2021–040).

### Statistical analysis

All assays were performed at least in triplicate and each experiment was repeated three times. All data were presented as the mean ± SEM, which was calculated from three independent experiments. Statistical significance among/between groups were examined with one-way analysis of variance (ANOVA) or independent-samples t-test. P < 0.05 was statistically significant.

## Result

### The effect of 3-Deoxysappanchalcone on microglia viability

**Fig 1:**
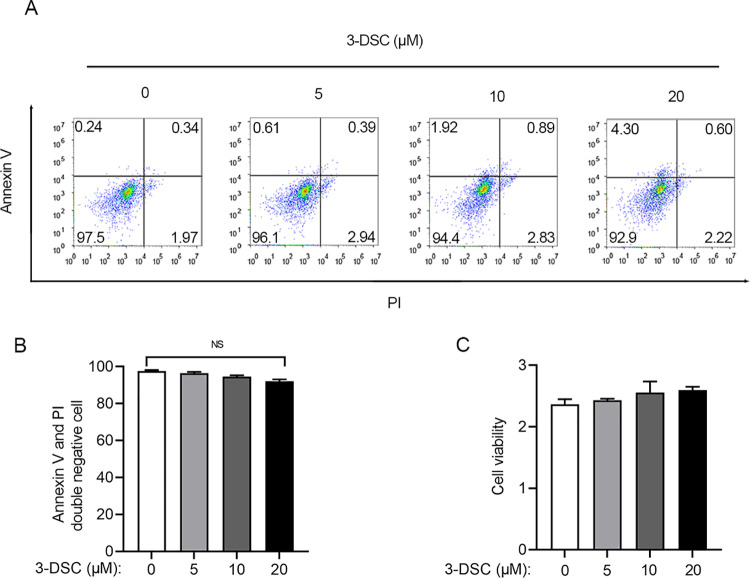
The effect of 3-DSC concentration on microglia apoptosis and viability. (A, B) Primary microglia were treated with 3-DSC at 0, 5, 10, and 20 μM for 12 h. The apoptosis rate of microglia in different group was assessed Annexin V/PI staining followed by flow cytometry analysis. Annexin V and PI double negative cells were regarded as normal cells. Representative flow cytometry results were shown in (A), the quantification of flow cytometry results was shown in (B). (C) Cell viability of primary microglia treated with 3-DSC at 0, 5, 10, and 20 μM for 12h was assay assessed by CCK8 assay. Data are shown as mean ± SEM of three independent experiments, ns, not significant, * P < 0.05, ** P < 0.01, *** P < 0.001.

To investigate the impact of 3-DSC on microglia-mediated inflammation, our initial step involved assessing how various concentrations of 3-DSC might influence microglia. We exposed primary microglia to varying concentrations of 3-DSC for 24 hours and conducted both flow cytometry and CCK8 assays to evaluate their response. Flow cytometry analysis revealed that concentrations of 3-DSC up to 20 μM did not trigger microglial apoptosis or cell death (Fig 1A and 1B). This suggests that within this range, 3-DSC does not exert cytotoxic effects on microglia. Furthermore, CCK8 assay results corroborated these findings, demonstrating that 3-DSC concentrations equal to or below 20 μM did not hinder microglial growth, as depicted in Fig 1C. Overall, these observations indicate that 3-DSC, at concentrations up to 20 μM, neither induces apoptosis nor inhibits the proliferation of microglia, laying a foundation for further investigations into its potential effects on microglia-mediated neuroinflammation.

### 3-Deoxysappanchalcone inhibited the activation of microglia during neuroinflammation

**Fig 2:**
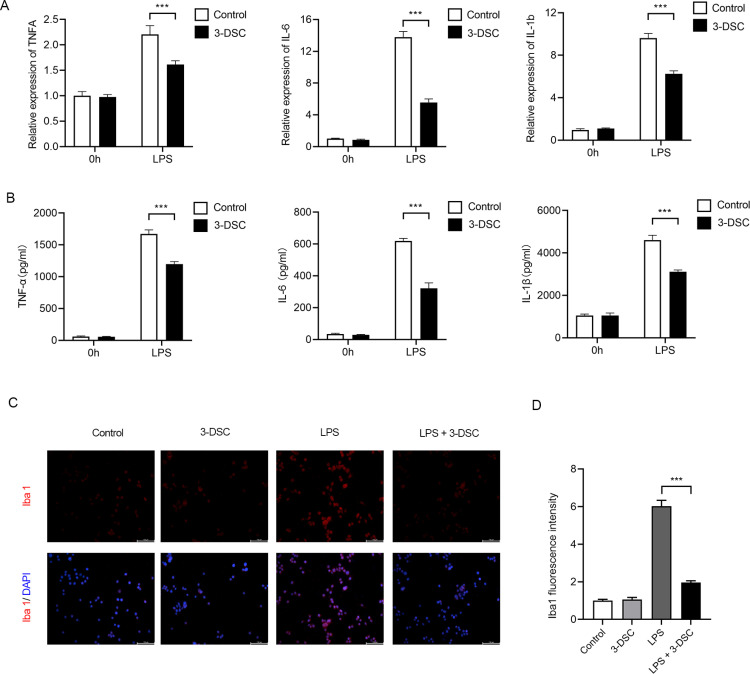
3-DSC inhibits the production of inflammatory cytokines in microglia. (A) qRT-PCR analysis of TNFA, IL-6, and IL-1b mRNA expression in microglia treated with 3-DSC followed by stimulation of LPS for 5h. (B) Elisa assay showing TNFα, IL-6 and IL-1β level in the supernatants from the cultures of microglia treated with 3-DSC followed by stimulation of LPS for 9h. (C, D) Representative images of immunofluorescence (C) staining for Iba1 (red) and quantification (D) of mean fluorescence intensity normalized to control. Data are shown as mean ± SEM of three independent experiments, ns, not significant, * P < 0.05, ** P < 0.01, *** P < 0.001.

The effect of 3-DSC was investigated on the expression of inflammatory cytokines. Microglia were treated with 3-DSC at a concentration of 10 μM one hour before being stimulated with LPS for 5 hours. The mRNA levels of TNF-α, IL-6, and IL-1β were measured via quantitative real-time PCR (qRT-PCR). The results indicated that 3-DSC significantly decreased the LPS-stimulated mRNA levels of TNF-α, IL-6, and IL-1β (Fig 2A). Furthermore, the results of the ELISA experiment were consistent with the qRT-PCR findings (Fig 2B).

Ionized Calcium Binding Adaptor Molecule 1 (Iba1) is one of the markers of microglia, and its expression represents the activation of microglia in neuroinflammatory states [19,20]. To investigate whether 3-DSC inhibits the activation of microglia, immunofluorescence experiments were performed. The results showed that the Iba1 fluorescence intensity of microglia was significantly decreased after 3-DSC treatment, indicating that 3-DSC could inhibit the activation of microglia (Fig 2C and 2D). Taken together, these data suggested that 3-DSC exerted an anti-inflammatory effect upon LPS stimulation in microglia.

### 3-Deoxysappanchalcone attenuated inflammatory signaling pathway activation

**Fig 3:**
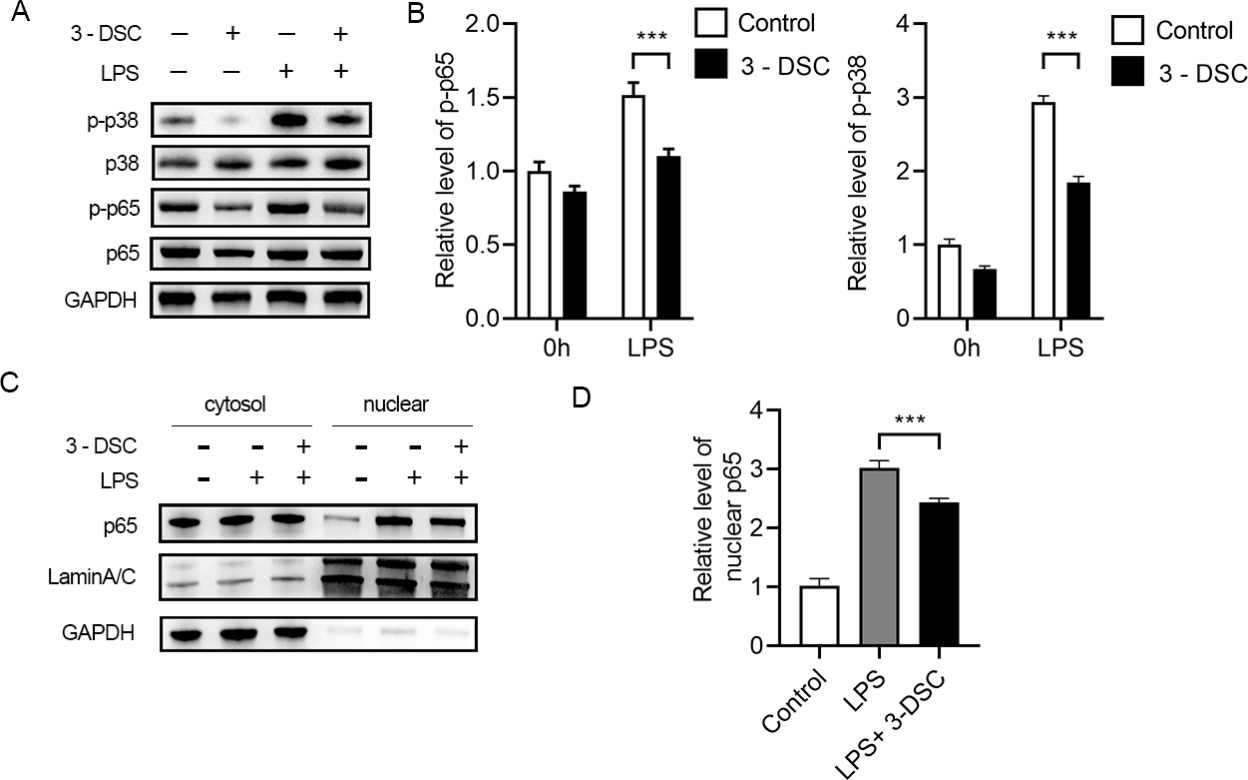
Inflammatory related signaling pathways in microglia are inhibited by 3-DSC. (A, B) Representative immunoblot (A) and the quantification (B) of phosphorylated (p-) proteins of microglia stimulated with LPS in the presence or absence of 3-DSC for indicated times. (C, D) Representative immunoblot (C) and the quantification (D) of nuclear p65 of microglia stimulated with LPS in the presence or absence of 3-DSC for indicated times. Data are shown as mean ± SEM of three independent experiments, ns, not significant, * P < 0.05, ** P < 0.01, *** P < 0.001.

To elucidate the mechanisms underlying the suppression of LPS-stimulated microglial pro-inflammatory response by 3-DSC, we initially investigated its impact on inflammatory signaling pathways, focusing on the canonical NF-κB and MAPK pathways [21,22]. Our results demonstrated that treatment with 3-DSC led to a reduction in the phosphorylation levels of p65 and p38 (Fig 3A and 3B). Furthermore, we examined the translocation of p65 and found that 3-DSC effectively inhibited its nuclear translocation (Fig 3C and 3D). Taken together, these findings indicate that 3-DSC can inhibit the activation of inflammation-related signaling pathways.

### 3-Deoxysappanchalcone inhibited proinflammatory genes and pathways under LPS stimulation

**Fig 4:**
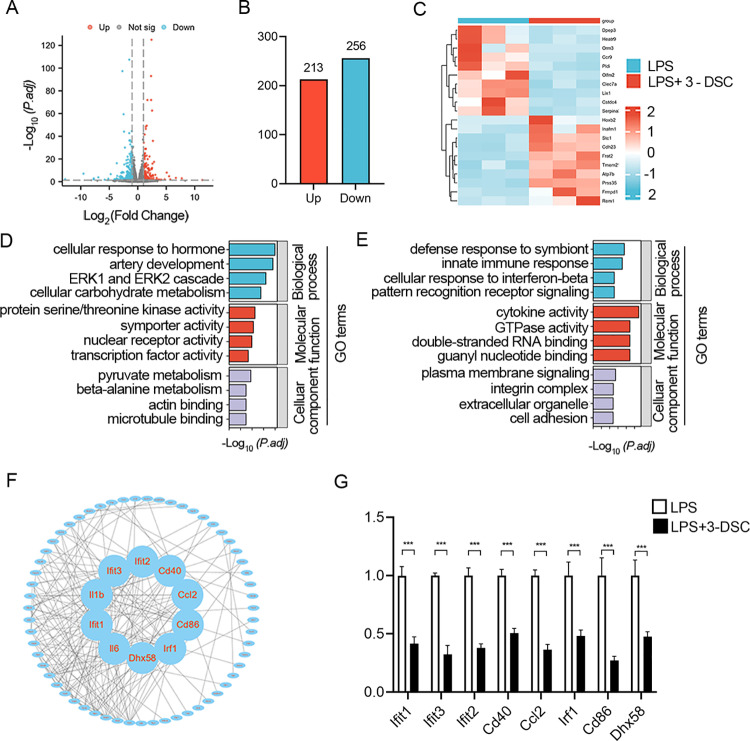
3-DSC treatment leads to changes in the expression of genes related to inflammatory responses. (A) Differential expression analysis reveals that 3-DSC induces transcriptional changes in microglia under LPS stimulation. Volcano plots show differentially expressed genes (DEGs; fold change≥2.0 or ≤−2.0, false-discovery-rate-adjusted P-value<0.05). (B) The number of DEGs is shown, a total of 213 upregulated genes and 256 downregulated genes were indicated. (C) The heatmap of the top 10 downregulated and upregulated genes significantly affected by 3-DSC treatment in microglia. (D, E) GO analysis showing the top four terms significantly affected by 3-DSC treatment, from the most upregulated (D) to the most downregulated (E). (F) PPI networks among the DEGs and hub genes as computed by Cytoscape. Connectivity degree levels were used to screen hub genes. (G) qRT–PCR analysis verified the expression of representative genes discovered by RNA-seq analysis after 3-DSC treatment. β-actin was used as an endogenous control. Data are shown as mean ± SEM of three independent experiments, ns, not significant, * P < 0.05, ** P < 0.01, *** P < 0.001.

To further study the function of 3-DSC in microglia, we comprehensively profiled the transcriptomic features between LPS-treated microglia and microglia treated with both LPS and 3-DSC by performing RNA-seq. Subsequently, we performed differential expression analysis to identify transcriptomic changes induced by 3-DSC A total of 469 differentially expressed genes (DEGs) were identified with the criteria of fold changes >2 or≤− 2 and P < 0.05. Among the 469 DEGs, there are 213 upregulated genes and 256 downregulated genes (Fig 4A and 4B). The heat map of the top 10 downregulated and upregulated genes influenced by 3-DSC is presented in Fig 4C. Gene ontology (GO) analysis revealed that downregulated gene-enriched GO terms were related to innate immune response and upregulated enriched GO terms were mostly associated with receptor activity (Fig 4D and 4E).

Subsequently, 469 DEGs were subjected to the STRING to predict protein interactions. We analyzed the PPI network and extracted the most highly connected cluster by MCODE plug-in in Cytoscape. The top 10 hub genes were IL-6, Ifit1, Ifit2, Ifit3, IL-1b, Cd40, Cd86, Ccl2, Irf1 and Dhx58(Fig 4F). Notably, all the 10 hub genes were related to inflammation process. Furthermore, qRT–PCR assays confirmed that the expression of all 10 genes was downregulated during treatment with 3-DSC (Fig 4G). These findings strongly indicate that 3-DSC systematically inhibits the inflammatory state of microglia.

### 3-Deoxysappanchalcone reduced activated microglia-induced apoptosis of neurons

**Fig 5:**
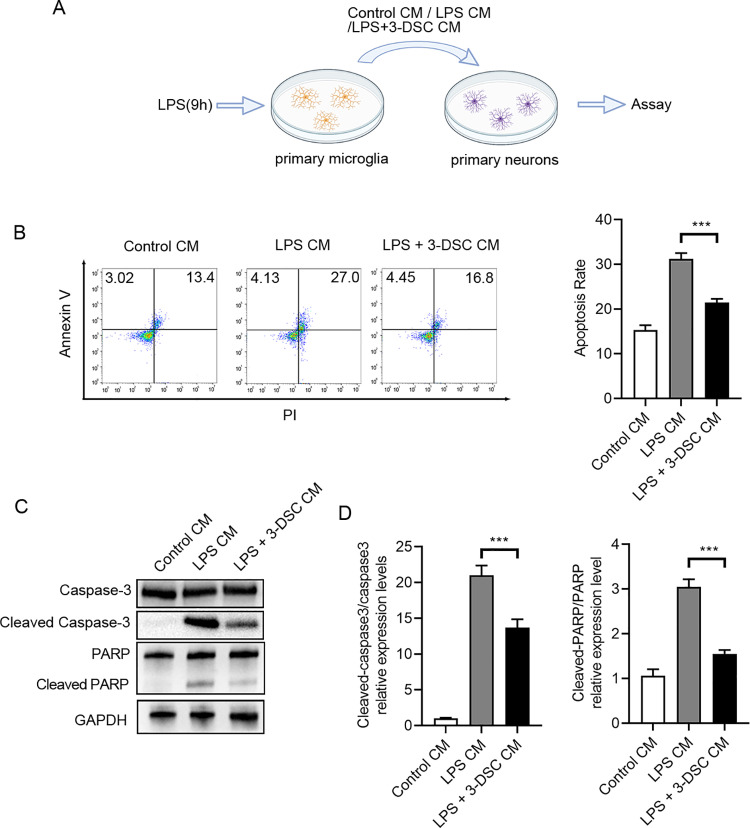
3-DSC reduces activated microglia-induced damage to neurons. (A) the schematic diagram of co-culture assays. Microglia were pretreated with 3-DSC for 1h and stimulated with 1 μg/mL LPS for 12h. Conditioned medium (CM) from microglia was collected and neurons were cultured in conditioned medium for 24 h. (B) the apoptosis rate of neurons in different group was assessed Annexin V/PI staining followed by flow cytometry analysis. Annexin V positive cells were regarded as apoptotic cells. (C, D) Representative immunoblot (C) and the quantification (D) of apoptosis-related proteins in neurons cocultured with different conditioned medium. Data are shown as mean ± SEM of three independent experiments, ns, not significant, * P < 0.05, ** P < 0.01, *** P < 0.001.

To investigate whether 3-DSC confers protective effects on neurons under inflammatory conditions, we conducted a coculture assay. The schematic diagram of coculture assay was shown in Fig 5A. Primary neurons were cultured in conditioned media obtained from microglia cell culture. Neurons treated with conditioned media from LPS-activated microglia exhibited a higher apoptosis rate compared to neurons plated in normal medium. However, treatment with 3-DSC specifically reduced the apoptosis rate of neurons in microglia-conditioned media (Fig 5B). Furthermore, a notable decrease in cleaved caspase-3 and cleaved PARP levels was observed in neurons exposed to conditioned media from 3-DSC-treated microglia (Fig 5C and 5D). These findings suggest that 3-DSC effectively reverses the apoptosis rate of neurons induced by LPS-stimulated microglia conditioned media.

### 3-Deoxysappanchalcone attenuated TBI-induced spatial memory impairment and microglia activation

**Fig 6:**
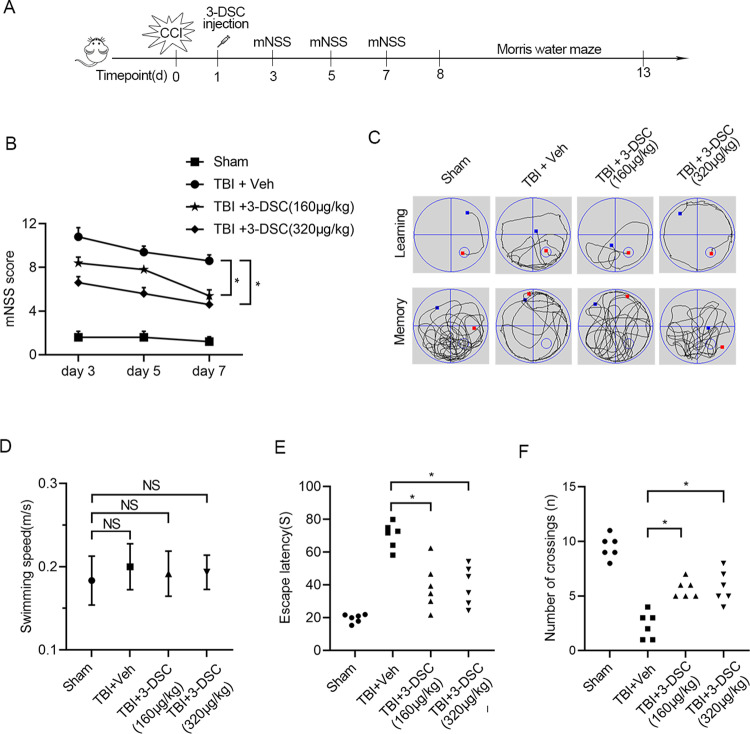
3-DSC ameliorate microglia activation, neurological dysfunction, and cognitive impairment induced by TBI. (A) Schematic diagram of the in vivo experimental protocols. (B) Neurological function between different group was evaluated by mNSS score. (C) Typical swimming paths in the Morris water maze during the training trials and probe trial. (D-F), swimming speed (E), escape latencies to find a hidden platform(E), and the frequency of crossing the hidden platform (F) were shown. Data are shown as mean ± SEM of three independent experiments, ns, not significant, * P < 0.05, ** P < 0.01, *** P < 0.001.

To explore whether 3-DSC plays an important role in TBI in vivo, we established TBI model with controlled cortical injury (CCI) in rats [23]. The study design was shown in Fig 6A. Briefly speaking, TBI rats were treated with 3-DSC (0.01 mol/kg and 0.02 mol/kg) and then followed with a variety of experiments. We first assessed the therapeutic effect of 3-DSC through modified neurological severity score (mNSS) [24]. As revealed by the mNSS, 3-DSC treatment following TBI promoted functional recovery (Fig 6B). Morris water maze test was conducted to evaluated spatial memory of TBI rats (Fig 6C) [25]. There was no significant difference in swimming speed between the different groups (Fig 6D). 3-DSC treatment facilitated spatial learning as suggested by reduced escape latencies during the training phase of the Morris water maze test compared to vehicle treated TBI model rats and better spatial memory as indicated by more former platform crossings (Fig 6E and 6F).

**Fig 7:**
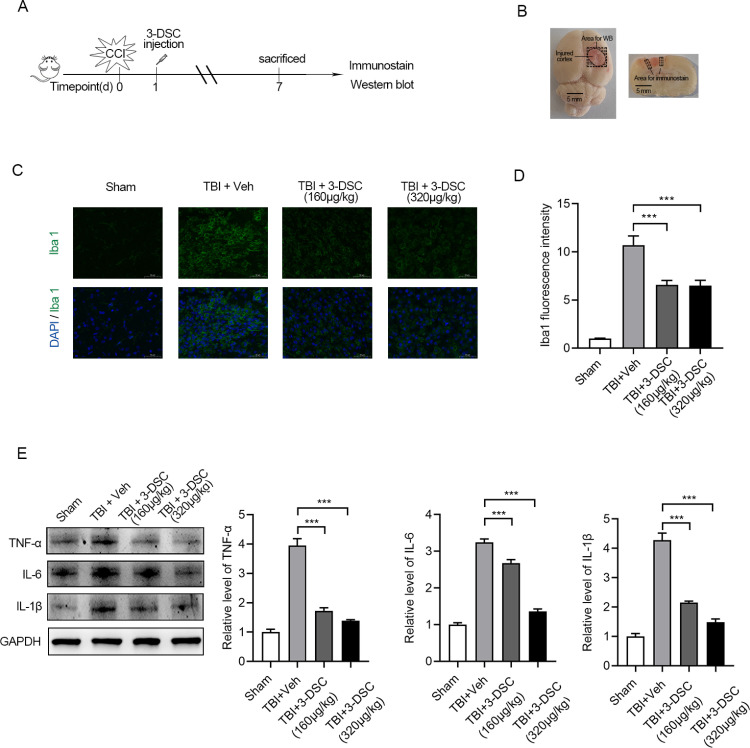
3-DSC ameliorate microglia activation and inflammatory cytokines production in vivo. (A, B) Schematic diagram of the in vivo experimental protocols (A) and gross observation of brain tissue(B) in CCI model. Scale bar = 3 mm. (C, D) Image panels of brain sections (C) immunolabeled for Iba1and quantification (D) of mean fluorescence intensity normalized to sham. (E, F) Representatives immunoblot (E) and the quantification (F) of TNF-α, IL-6 and IL-1β of brain tissues with different treatment. Data are shown as mean ± SEM of three independent experiments, ns, not significant, * P < 0.05, ** P < 0.01, *** P < 0.001.

Next, to detect inflammatory activation levels in brain tissue, brain tissues were collected and prepared for subsequent western blot and immunofluorescence (Fig 7A and 7B). Iba1 immunofluorescence was conducted to evaluate the activation of microglia. 3-DSC treatment inhibited Iba1 expression levels in microglia, compared to that in the vehicle group (Fig 7C and 7D). Consistent with the previous in vitro results, western blot results confirmed that administration of 3-DSC significantly reduced TNF-α, IL-6 and IL-1β expression levels in the injured cortex (Fig 7E and 7F). Taken together, 3-DSC treatment significantly attenuated microglia activation and promoted cognitive recovery induced by TBI.

## Discussion

Traumatic brain injury ranks as a leading cause of death, with estimates ranging from 27 to 69 million incidences annually worldwide, resulting in over 1 million deaths [26]. TBI results in approximately 8.1 million people worldwide becoming disabled yearly due to TBI and incurs an annual healthcare cost of approximately $400 billion [27,28]. Consequently, TBI poses significant concerns for affected individuals, their families, healthcare professionals, and society at large.

The pathology of traumatic brain injury (TBI) is intricate, encompassing both immediate and delayed damage mechanisms. The primary injury, stemming from the initial impact, is deemed irreversible. Subsequently, the secondary phase unfolds over hours to days, and sometimes months post-injury, presenting a critical opportunity for therapeutic intervention [29,30]. The significance of neuroinflammation and vascular pathology, particularly blood-brain barrier (BBB) dysfunction, in driving brain injury and neural degeneration after TBI has been well recognized [31,32]. Prolonged disruption of the blood-brain barrier initiates a cascade of events culminating in excessive neuroinflammation. The leakage of serum proteins, notably albumin, which is abundant in blood, induces glial cells to enter a pro-inflammatory state [33]. These activated glial cells then release various cytokines such as IL-6, IL-1β, and TNF [34,35]. This neuroinflammatory response exacerbates BBB permeability, further compromising its integrity.

Derived from Caesalpinia sappan L, 3-DSC is a compound with multiple effects including antioxidant, anti-inflammatory, and anti-cancer properties [9,36]. However, its potential to inhibit neuroinflammation has not yet been reported. In our experiments, to investigate the potential anti-inflammatory effects of 3-DSC, a rat model of TBI and an in vitro neuroinflammation model of LPS-stimulated microglia, which are currently widely used as disease models, were constructed. Our in vitro experimental results indicated that 3-DSC can inhibit the secretion of IL-6, IL-1β, and TNF by microglia. These findings suggest that 3-DSC has the function of suppressing neuroinflammation. In terms of signaling pathways, we found that 3-DSC can inhibit some classical inflammatory signaling pathways, such as NF-κB and MAPK [22,37], indicating that its anti-inflammatory effects are partially mediated through these pathways. To further investigate how 3-DSC affect microglial cells, we conducted transcriptomic sequencing of microglial cells. RNA-seq results showed that the downregulated gene-enriched GO terms were also related to interferon (IFN) signaling (Fig 3E). Given that IFN-related genes are strongly induced by TBI and blocking the IFN response can benefit neuroinflammation and functional recovery, the beneficial effects of 3-DSC on TBI warrant more thorough investigation [38,39]. Under the condition of neuroinflammation, neurons are prone to undergo the process of apoptosis [40]. The direct action of inflammatory mediators plays a crucial role [41]. To simulate the inflammatory state, we used a co-culture model, which is one of the commonly used models for neuroinflammation. The cell supernatant of LPS-stimulated microglia contains many inflammatory factors [42]. Using this supernatant to culture neuron cells can simulate the neuroinflammation state. We found that 3-DSC pretreatment on the activated microglia significantly increased neuron viability by inhibiting apoptosis induced by an activated microglia-conditioned medium. This result proved that 3-DSC has a favorable anti-inflammatory effect and can alleviate the apoptotic state of neurons under the neuroinflammation.

The CCI model is a widely utilized and highly regarded experimental paradigm in the field of TBI [43,44]. It employs a precisely calibrated device that delivers a defined impact force to the cerebral cortex of experimental animals, typically rodents. By precisely controlling parameters such as impact velocity, depth, and duration, researchers can induce reproducible and graded levels of brain injury which could accurately mimic the real-world TBI cases. In the subsequent days and weeks following injury, the neuroinflammation characterized by the activation of microglia and astrocytes and the release of inflammatory mediators progresses over time. Following TBI, significant behavioral changes can be observed [45]. In the initial days post-injury, animals or patients may display disorientation and confusion. Motor impairments are also prevalent. Coordination and balance problems are frequently seen. Fine motor skills, such as grasping objects or performing delicate tasks, are severely compromised [46]. Cognitive deficits are another hallmark of TBI-induced behavioral changes. Memory problems, especially short-term memory loss, are common [47]. Morris water maze and mNSS are frequently used for the prognostic analysis of neurological injuries. The Morris water maze is a behavioral test paradigm that has been invaluable in assessing spatial learning and memory capabilities [48,49]. mNSS is a comprehensive neurological assessment scale. It encompasses a range of motor, sensory, and reflex functions. Each function is scored based on a set of predefined criteria, and the overall score provides an indication of the severity of neurological impairment. Our results showed that during the 14-day functional recovery phase, the 3-DSC group rats had more crossing and rearing numbers than the sham group rats, thus implying that both motor function and exploratory ability were restored to a certain extent in the 3-DSC group. The neurological function scores of the 3-DSC group were better than those of the CCI group. These results suggest that 3-DSC can effectively alleviate TBI and promote the recovery of histological and motor functions in rats. The immunofluorescence experiment results around the injured tissue indicate that 3-DSC inhibits microglial cell activation. Furthermore, Western blot results indicate that the levels of inflammatory factors in the injured tissue are reduced in the 3-DSC group. All these findings suggest that 3-DSC inhibits the inflammatory levels in the brain tissue of traumatic brain injury (TBI) rats.

In conclusion, we reveal a novel role for 3-DSC in inhibiting microglia-mediated neuroinflammation. 3-DSC is a promising therapeutic target of neuroinflammation. For clinical applications, we provide theoretical support for the effect of 3-DSC, but more in-depth clinical trials are needed.

## Supporting information

S1 Dataset The complete raw data (minimal data set) for all gels and blots in this manuscript can be found in the article’s supplementary data. (RAR)
